# Random sampling of the Protein Data Bank: *RaSPDB*

**DOI:** 10.1038/s41598-021-03615-y

**Published:** 2021-12-17

**Authors:** Oliviero Carugo

**Affiliations:** 1 Department of Chemistry, University of Pavia, Viale Taramelli 12, 27100 Pavia, Italy; 2 Department of Structural and Computational Biology, University of Vienna, Vienna, Austria

**Keywords:** Biochemistry, Computational biology and bioinformatics, Molecular biology, Structural biology

## Abstract

A novel and simple procedure (RaSPDB) for Protein Data Bank mining is described. 10 PDB subsets, each containing 7000 randomly selected protein chains, are built and used to make 10 estimations of the average value of a generic feature F—the length of the protein chain, the amino acid composition, the crystallographic resolution, and the secondary structure composition. These 10 estimations are then used to compute an average estimation of F together with its standard error. It is heuristically verified that the dimension of these 10 subsets—7000 protein chains—is sufficiently small to avoid redundancy within each subset and sufficiently large to guarantee stable estimations amongst different subsets. RaSPDB has two major advantages over classical procedures aimed to build a single, non-redundant PDB subset: a larger fraction of the information stored in the PDB is used and an estimation of the standard error of F is possible.

## Introduction

The Protein Data Bank (PDB) is the largest collection of macromolecule three-dimensional structures determined experimentally with X-ray crystallography, cryo-EM, NMR, and with other, relatively uncommon techniques^1,2^. It has countless applications: it is in the PDB that crystallographers find the information necessary to solve the phase problem with molecular replacement methods^3^; bioinformaticians find there templates for homology modelling of protein structures^4^; drug discovery has been greatly influenced by PDB too^5^; and the information deposited in the PDB has been widely used to delineate structural trends and prediction tools, dealing with secondary structure^6^, protein motifs^7^ or accessibility to internal, buried cavities^8^, just to mention few examples.

For estimating any feature Φ from the PDB it is necessary to build a non-redundant subset of the PDB^9^. This is generally performed by selecting an arbitrary maximum percentage of sequence identity (PMAX), usually in the 20–40% range, so that the resulting subset contains only entries with percentages of sequence identity lower than PMAX^10,11^. This is necessary, since the PDB is known to be rather redundant^12^ and, as a consequence, only biased estimations of any Φ can be determined from it.

Although the redundancy limitation is a well-accepted procedure, it has two main drawbacks; it reduces, sometime enormously, the amount of information stored in the PDB and it does not allow the estimation of the standard error of Φ.

An alternative procedure, resembling a blocking factor analysis^13^ and referred to as *RaSPDB*, is described in the present communication: *N* equally populated subsets of the PDB, each containing *D* protein chains, are randomly assembled, as in blocking factor analysis.

An evident advantage of *RaSPDB* is that the value of Φ can be computed *N* times, each time being independent of the others, and that it is consequently possible to compute the average value of the *N* values of Φ together with its standard error and, if needed, other figures of merit related to the distribution of the *N* values of Φ. This is impossible by following the traditional procedure, where a single subset of the PDB is built and used as a unique source of information.

Moreover, this *RaSPDB* allows the use of a larger amount of the information stored in the PDB.

A possible problem of *RaSPDB* is the redundancy level within the *N* subsets containing *D* files. However, if *D* is sufficiently small relative to the dimension of the PDB, this is clearly a minor problem, since the probability to pick-up homologous entries is very small. On the contrary, if *D* is large, it would be not surprising to find homologous entries in the same subset.

Apropos of redundancy, small *D* values—which means small subsets of the PDB—are thus advisable. However, if *D* is too small it is possible that the estimations of Φ are extremely unstable from one subset to the next. In other words, it is impossible to obtain reliable estimations of Φ if sampling is insufficient. For this reason, the dimension of the subsets cannot be too small.

It is consequently necessary to attain a compromise solution, in which *D* must be small enough to avoid excessive redundancy and big enough to ensure sufficient sampling.

Worthy to be remembered, eventually, that RaSPDB cannot substitute additional criteria for assembling high quality subsets of the PDB, like R-factor, free-R-factor, crystallographic resolution and others^9^. It can, however, flank these strategies for improving PDB mining.

## Results and discussion

### Random sampling

In the present communication, the number of subsets is fixed to *N* = 10 and the dimension of each subset is varied from 100 to 10,000 (*D* = 100, 500, 1000, 2000, 3000, 4000, 5000, 6000, 7000, 8000, 9000 or 10,000). The random assembly of the subset was performed as described in the Supplementary Material.

### Φ stability

A series of features Φ were examined, some related to sequence (the length of the protein chain defined by its number of residues, and the amino acid composition) and others related to structure (the crystallographic resolution and the secondary structure composition).

*N* average values were calculated for the *N* subsets. For example, the length of the protein chain (*#aa*) was computed in all the *D* sequences of an individual subset, these *D* values were averaged within each individual subset, and then the *N* average values of *#aa* were analyzed: the minimum, maximum and average values were determined together with the estimated standard error of the mean.

**Figure 1:**
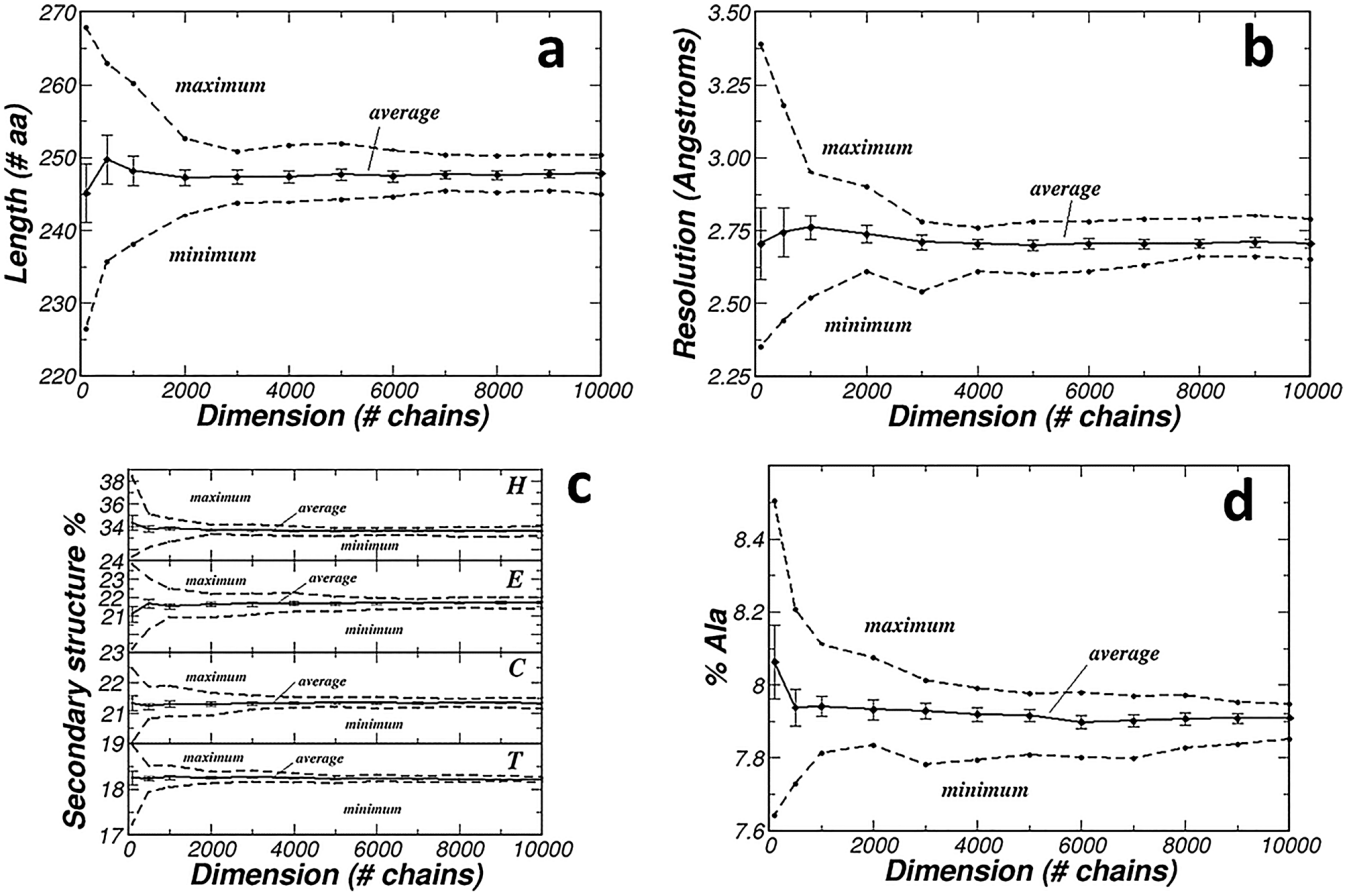
Optimization of the dimension of the subsets. (**a**) Relationship between protein chain length and dimension of the randomly selected subsets of the PDB. (**b**) Relationship between resolution and dimension of the randomly selected subset of the PDB. (**c**) Relationship between secondary structure composition and dimension of the randomly selected subsets of the PDB (secondary structures were assigned with STRIDE^14^, and only the most common H (α-helix), E (β-strand), C (coil), and T (turn) types of secondary structure were considered. (**d**) Relationship between amino acid composition and dimension of the randomly selected subsets of the PDB (only the case of alanine in shown, being the plots relative to other amino acids very similar).

Figure 1a shows how the length of the protein chain depends on *D*. As expected, if *D* is small (for example *D* = 100), the average protein length is quite variable amongst the *N* = 10 subsets. It ranges from 226.5 to 267.9 with an average value of 245.1 and an estimated standard error of 4.0. On the contrary, if *D* is larger (for example *D* = 10,000), the average protein length is much less variable amongst the *N* = 10 subsets. The difference between the maximal (250.3) and minimal (244.9) values is much smaller and the average value (247.8) is associated with a much smaller estimated standard error (0.5).

The difference between the maximal and minimal value of protein length decreases when *D* increases, up to *D* = 6000. It goes from 41.4 to 27.2, 22.1, 10.6, 7.9, 7.1, 7.7 and 6.4 when *D* goes from 100 to 500, 1000, 2000, 3000, 4000, 5000 and 6000. Then is close to 5 and nearly constant for larger D values (4.9 for *D* = 7000, 5.0 for *D* = 8000, 4.9 for *D* = 9000 and 5.4 for *D* = 10,000).

The estimated standard errors decrease too when *D* increases, going from 4.0 for *D* = 100 to 0.74 for *D* = 6000. Then, for larger *D* values, they are nearly constant (0.57 for *D* = 7000, 0.61 for *D* = 8000, 0.52 for *D* = 9000 and 0.52 for *D* = 10,000).

Note that both protein length average values and standard errors are computed on the ten average values, each of which is computed with one of the ten subsets of size *D*. The decrease of the standard errors is thus not due to the growing of the number of data used to computed them.

This trend is observed not only when Φ is the length of the protein chain. Figure 1b–d show the dependence on *D* of other features, the crystallographic resolution, the secondary structure composition, and the amino acid composition.

By visual inspection it is possible to estimate that, *on average*, the divergence between the estimations of Φ in different subsets of the PDB decreases up the *D* = 6000 to become stable for larger subsets with *D* ≥ 7000.

It is thus reasonable to deduce that subsets of the PDB containing 7000 protein chains are large enough for sampling the corpus of protein structures deposited in the PDB.

### Internal redundancy

It is now necessary to check the level of redundancy in randomly selected subsets of the PDB that contain 7000 protein chains.

Given that an all-against-all sequence alignments would have been too expensive—nearly 25 million alignments for each of the ten PDB subsets—only 10,000 randomly selected alignments were considered for each subset.

The average redundancy is very small. Amongst the ten PDB subsets: the average pairwise percentage of sequence identity ranges from 9.74 to 11.18%, with a mean value of 10.52% (estimated error 0.06%).

The percentage of sequences pairs with percentage of sequence identity larger than 40% is also small (0.06%).

This clearly shows that randomly built PDB subsets containing 7000 protein chains are sufficiently small to avoid excessive internal redundancy.

Furthermore, it is necessary to check how frequently the same protein sequence is randomly selected in two or more of the 10 PDB subsets. In other words, it is necessary to evaluate the degree of overlap between the subsets. This is a relevant problem, since there are 70,000 entries in ten groups of 7000 entries and this represents a considerable fraction (about 40%) of the whole PDB.

The overlap between the two groups, each containing *D* protein sequences, is evaluated by removing duplicate sequences from one of them. As a consequence, two groups might contain *R* and *R’* protein sequences, with *R* ≤ *D* and *R′* ≤ *D*. The expression *NOVP* = 100 min(*R*,*R′*)/*D* would be equal to 100 if no overlap occurs, and it would be lower than 100 in case of overlap. Here *NOVP* ranges from 66 to 100, amongst all unique pairs of the ten PDB random subsets, with an average vale of 81 (standard error 3). This indicates that it is uncommon that the same structure is selected twice in ten PDB subsets of 7000 protein chains.

## Conclusions

It is thus possible to sample the Protein Data Bank by constructing several subsets, the elements of which are selected randomly. The reason why this is possible is essentially due to the large dimension of the Protein Data Bank—about 180,000 entries.

This approach allows repeating the same estimation or analysis several times, each time with one of the subsets, and this allows, consequently, assessing the degree of reliability of the estimation/analyses.

It is however necessary to mention an additional and very important issue. It is well known that the degree of reliability is not distributed uniformly in the Protein Data Bank: some structures are more reliable than others are, and even different parts of the same structure may have different reliabilities^15^. Consequently, analyses and surveys of the Protein Data Bank are performed on smaller subsets that are selected according to various criteria, which depend on the type of analysis/survey that must be done. The strategies to build these subsets of the Protein Data Bank have been recently reviewed^9^ and the Protein Data Bank provides a validation report for each entry (http://www.wwpdb.org/validation/validation-reports).

It is recommendable therefore to, first, eliminate the PDB entries that do not fulfill the quality criteria, for example all non-crystal structures and crystal structures refined at insufficient resolution; and only after that to apply the RaSPDB strategy for building subsets, the elements of which are randomly selected. In this way, it is possible to ensure both data quality and effective sampling.

A limitation of this sampling strategy must be noticed. It cannot be used for small ensembles of data, for example, when it is necessary to consider only protein crystal structures refined at an extremely high resolution, better than 1.0 Å (about 800 entries) or protein crystal structures determined at a temperature lower than 70 K (about 200 entries).

## Methods

A file containing sequences in FASTA format for all entries in the PDB archive was downloaded from http://www.rcsb.org/downloads/fasta. After removing all non-protein sequences, N groups containing D protein chains—with N = 10 and D = 100, 500, 1000, 2000, 3000, 4000, 5000, 6000, 7000, 8000, 9000 or 10,000—were randomly extracted (see Supplementary Material).

Secondary structures were assigned with STRIDE^14^, lengths of protein chains were determined from the SEQRES lines of the PDB files. Sequence alignments were performed with the Needleman-Wunsch algorithm, coded into the EMBOSS routine Needle^16^. All statistical analyses were performed with locally written software.

## Supplementary Information

Supplementary Information.
