# Supplementary material for: Random sampling of the Protein Data Bank: RaSPDB
Source: Sci Rep. 2021 Dec 17;11:24178. doi: 10.1038/s41598-021-03615-y (PMC8683422; doi:10.1038/s41598-021-03615-y)
Supplement: Supplementary file 1 — Supplementary Information. [file 41598_2021_3615_MOESM1_ESM.docx]

Random sampling of the Protein Data Bank

**Oliviero Carugo**

*Department of Chemistry, University of Pavia, Pavia, Italy and Department of Structural and Computational Biology, University of Vienna, Vienna, Austria (ORCID 0000-0002-2924-9016)*

Correspondence to:

Oliviero Carugo

Department of Chemistry

University of Pavia

Viale Taramelli 12

I-27100 Pavia, Italy

Email: [Oliviero.carugo@univie.ac.at](mailto:Oliviero.carugo@univie.ac.at)

# Supplementary Material

# Random assembly of the subsets

A file containing sequences in FASTA format for all entries in the PDB archive can be downloaded from http://www.rcsb.org/downloads/fasta. After removing all non -protein sequences, N groups containing D protein chains - with N = 10 and D = 1 00, 500, 1000, 2000, 3000, 4000, 5000, 6000, 7000, 8000, 9000 or 10000 - are randomly extracted by using functions of the C programming language. No more than one chain per PDB entry was included in one group.
